# Mass Spectrometric-Based Selected Reaction Monitoring of Protein Phosphorylation during Symbiotic Signaling in the Model Legume, *Medicago truncatula*

**DOI:** 10.1371/journal.pone.0155460

**Published:** 2016-05-20

**Authors:** Lori K. Van Ness, Dhileepkumar Jayaraman, Junko Maeda, Gregory A. Barrett-Wilt, Michael R. Sussman, Jean-Michel Ané

**Affiliations:** 1 Department of Biochemistry, University of Wisconsin–Madison, Madison, WI, 53706, United States of America; 2 Department of Agronomy, University of Wisconsin–Madison, Madison, WI, 53706, United States of America; 3 Department of Bacteriology, University of Wisconsin-Madison, Madison, WI, 53706, United States of America; 4 Mass Spectrometry/Proteomics Facility, University of Wisconsin–Madison, Madison, WI, 53706, United States of America; 5 Biotechnology Center, University of Wisconsin–Madison, Madison, WI, 53706, United States of America; Centre National de la Recherche, FRANCE

## Abstract

Unlike the major cereal crops corn, rice, and wheat, leguminous plants such as soybean and alfalfa can meet their nitrogen requirement via endosymbiotic associations with soil bacteria. The establishment of this symbiosis is a complex process playing out over several weeks and is facilitated by the exchange of chemical signals between these partners from different kingdoms. Several plant components that are involved in this signaling pathway have been identified, but there is still a great deal of uncertainty regarding the early events in symbiotic signaling, i.e., within the first minutes and hours after the rhizobial signals (Nod factors) are perceived at the plant plasma membrane. The presence of several protein kinases in this pathway suggests a mechanism of signal transduction via posttranslational modification of proteins in which phosphate is added to the hydroxyl groups of serine, threonine and tyrosine amino acid side chains. To monitor the phosphorylation dynamics and complement our previous untargeted 'discovery' approach, we report here the results of experiments using a targeted mass spectrometric technique, Selected Reaction Monitoring (SRM) that enables the quantification of phosphorylation targets with great sensitivity and precision. Using this approach, we confirm a rapid change in the level of phosphorylation in 4 phosphosites of at least 4 plant phosphoproteins that have not been previously characterized. This detailed analysis reveals aspects of the symbiotic signaling mechanism in legumes that, in the long term, will inform efforts to engineer this nitrogen-fixing symbiosis in important non-legume crops such as rice, wheat and corn.

## Introduction

Most plants belonging to the legume family utilize symbiotic associations with nitrogen—fixing bacteria called rhizobia, to meet their nitrogen requirement [1, 2]. In this mutualistic association, rhizobia reside in specialized organs called nodules that are formed in the plant roots, where they fix atmospheric dinitrogen into plant-available ammonia, in exchange for a carbon source [1, 3]. This interaction is highly specific and is facilitated by the exchange of chemical signals between plant and microbes. The perception of flavonoids and isoflavonoids that are released by legume roots triggers the expression of *nod* genes in rhizobia, leading to the production of lipo-chito-oligosaccharidic molecules called Nod factors (NF) [4, 5]. Plant host specificity is governed by these bacterially produced NFs, which are active at extremely low concentrations (10^−8^ to 10^−12^ M). Purified NF alone is capable of triggering host plant responses, akin to rhizobia themselves, and are often used to study early events in symbiotic signaling [4, 6]. For instance, some responses, such as plasma membrane ion fluxes, cytoplasmic alkalinization and reactive oxygen species accumulation, occur within a few seconds or minutes after NF application, whereas periodic oscillations in calcium concentrations in and around the nucleus, calcium spiking, occurs approximately 15 to 20 min after NF application, leading to early nodulin gene expression [6–8]. In addition, root hair swelling followed by root hair deformation also occurs within one hour of NF application [9, 10].

Research on the model legumes *Medicago truncatula* and *Lotus japonicus* using genetic, transcriptomic and biochemical approaches has led to the identification of several components that are involved in this symbiotic signaling cascade [2]. These model legumes, in addition to forming a symbiotic association with bacteria, also form an association with fungi called arbuscular mycorrhizal fungi and certain genes called ‘common symbiotic genes’ are indispensable for both of these symbioses [1, 11]. Using loss-of-function mutants for root nodule and/or arbuscular mycorrhizal symbioses, several symbiotic genes have been cloned, and analyses of these mutants have helped to establish a genetic hierarchy of the genes [12, 13]. LysM-domain-containing receptor-like kinases have been implicated in the binding of NF, and in *M*. *truncatula*, mutants of Nod Factor Perception (NFP) are defective in all known early responses to NF, including root hair deformations, calcium spiking, and early nodulin expression. Therefore, NFP is placed at the top of the symbiotic signaling cascade [14, 15]. Although direct experimental evidence is lacking, it is widely believed that the leucine-rich receptor-like kinase Does not Make Infections 2 (DMI2) acts as a co-receptor for NF. In addition to the absence of a calcium-spiking phenotype, *dmi2* mutants are also impaired in rhizobial infection. Because these mutants exhibit certain early responses to NF, such as root hair swelling and branching, they are placed downstream of NFP in the symbiotic signaling cascade [13, 16, 17]. The signals that are perceived by these plasma membrane receptor-like kinases are transduced to the ion channel Does not Make Infections 1 (DMI1), which is localized on the nuclear membrane [18–20]. Although it is still unclear how the signals are transduced to DMI1, it has been hypothesized that mevalonate or its products may act as secondary messengers in this signal transduction [21]. Furthermore, posttranslational modifications, such as phosphorylation, may also play a major role in transducing signals from the plasma membrane to the nucleus. The perception of signals by DMI1 leads to calcium spiking in the peri-nuclear and nuclear regions. Both bacterial and AM fungal symbioses elicit calcium spiking, which varies in the frequency and amplitude of the spikes [22]. Encrypted information in the calcium spikes are decoded by the calcium/calmodulin-dependent protein kinase (CCaMK) Does not Make Infections 3 (DMI3) [23–25]. Mutants of DMI3 are impaired in early nodulin gene expression and root nodule formation but undergo earlier events, such as root hair deformations and calcium spiking [13, 23]. Downstream of DMI3, several transcription factors are activated, leading to gene expression, which ultimately culminates in the formation of nodules, or vesicles and arbuscules for arbuscular mycorrhization [2].

Despite the identification of several components of the symbiotic signaling cascade, there are still missing links, particularly in the early stages [21]. To dissect the symbiotic signaling cascade more precisely, several high-throughput transcriptomic, proteomic, proteogenomic and metabolomic approaches have been adopted [26, 27]. With the exception of a few studies [28–30], most approaches used late time points after inoculation with rhizobia or NF. In our previous study [26], we performed discovery proteomics experiments that identified 136 genes and 98 phosphoisoforms that were differentially regulated very early in the response, *i*.*e*. one hour after NF treatment. The discovery data were acquired using isobaric-tagged peptides that were analyzed after SCX fractionation and IMAC phosphopeptide enrichment on an ETD-enabled Orbitrap Velos hybrid linear ion trap-Orbitrap mass spectrometer. In this untargeted (discovery) method only the most abundant peptides are selected for further sampling [31]. The advantage of this method is that no prior knowledge of the peptide sequence is required. A challenging disadvantage, however, is a decreased ability to routinely and predictably detect lower-abundance peptides [31, 32]. To overcome this, we applied a targeted approach known as SRM (Selected Reaction Monitoring) that utilizes synthetic heavy—isotope—labeled tryptic peptides as internal standards, with a triple quadrupole mass spectrometer. This method enables us to perform a high-throughput and reliable analysis of changes in the phosphorylation pattern of specific peptides, implicated in the earlier Nod factor signaling discovery study. Utilizing SRM, we have thus validated the NF-dependent phosphorylation of candidate proteins identified previously to provide insights into missing parts of the symbiotic signaling cascade.

## Materials and Methods

### Plant growth and sample preparation

Seeds of wild-type *M*. *truncatula* Jemalong cv A17 (wild-type), *nfp* mutant C31, *dmi2* mutant TR25 and *dmi3* mutant TRV25 were acid scarified, surface sterilized and germinated as described in [33]. Overnight-germinated seedlings were grown in the dark for 5 days on nitrogen-free Fahraeus medium that was overlaid with sterile germination paper as in Rose *et al*., 2012. Then, 5-day-old seedlings were flood inoculated with 10^−8^ M Nod factors (NF) from *Sinorhizobium meliloti*, a symbiont of *Medicago truncatula*, such that all of the roots were covered. As a control, mock-treated plants were used. Various time points, such as 5 min, 15 min, 30 min and 60 min, were used for wild-type seedlings to understand the phosphorylation dynamics of the selected peptides. In addition, the *nfp* and *dmi2* mutants were NF-treated for 15 min and the *dmi3* mutant was NF-treated for 15 min and 60 min. Each treatment consisted of five replicates per condition, i.e., five mock-treated plates and five NF-treated plates. The seedlings were treated for the specified time, harvested, immediately flash frozen in liquid nitrogen, and ground with mortar and pestle. The samples were further homogenized in grinding buffer (290 mM Sucrose, 250 mM Tris-HCl pH 8, 25 mM EDTA, 50 mM Sodium Pyrophosphate, 25 mM Sodium Fluoride, 1 mM ammonium molybdate, and 0.5% w/v polyvinylpyrrolidone) that was supplemented with freshly added phosphatase and protease inhibitors (2 mM Vanadate, 0.1 mM phenanthroline, 1 μg/ml E64, 10 μg/L leupeptin, 1 mg/L pepstatin, 1 μM bestatin, 1 mM PMSF, and 1 mM dithiothreitol) using sonication (1-cm probe, 30 s × 4, and 50% duty cycle) while kept on ice. The resulting homogenate was filtered through four layers of Miracloth (Calbiochem, Billerica, MA) and spun (16,000 × *g*, 20 min, and 4°C) to remove debris.

### Targeted SRM analysis

Proteins were precipitated from sample supernatant with acetone overnight at 20°C by adding four volumes of ice-cold acetone to one volume of plant extract. The resulting precipitate was resuspended in 2% SDS, 1 mM dithiothreitol, Tris-HCl pH 8 buffer prior to methanol/chloroform/water protein extraction. Methanol/chloroform extraction was performed by adding four volumes of methanol to one volume of resuspended plant extract. The mixture was vortexed, and one volume of chloroform was added before additional vortexing. Three volumes of water were then added, and the solution was vortexed and subsequently centrifuged for 15 min (2500 × *g*, 20°C). The top layer was removed and discarded, retaining the interphase region. Then, three volumes of methanol were added, and the solution was vortexed and centrifuged. Pellets were washed with 80% acetone. The extracted protein was resuspended in 6 M urea and 1x phosSTOP (Roche, Indianapolis, IN) and quantified using a bicinchoninic acid assay kit at 1:10 and 1:20 dilutions per sample (Pierce, Grand Island, NY).

For each sample, 5 mg of protein was spiked with an identical volume (30μl) of pooled, isotopically labeled phosphorylated phosphopeptide standards (see below, synthesized by the University of Wisconsin—Madison Biotechnology Center’s peptide synthesis core facility or Sigma Aldrich, St. Louis, MO, and of at least 90% purity). The labeled phosphopeptide standards were mixed at varying concentrations chosen to yield a consistent signal level for the primary SRM transition for each peptide. The samples were reduced with 5 mM dithiothreitol (30 min at 55°C) and alkylated using 15 mM iodoacetamide (30 min at room temperature). The samples were digested for two hours at 37°C in 6 M urea using Lys C (Wako) at a 1:100 enzyme: protein ratio, then diluted to 1.5 M urea using 20 mM Tris-HCl pH 8 and further digested with trypsin (Promega, Madison, WI) at a 1:250 enzyme:protein ratio overnight at 37°C. The samples were acidified to 0.5% v/v TFA to stop enzymatic digestion and desalted using C18 solid-phase extraction columns (Waters, Milford, MA).

Phosphopeptide enrichment was performed using homemade TiO_2_ columns containing 4 mg of TiO_2_ particles (10 μm; GL Sciences, Torrance, CA), as described by Minkoff *et al*. (2014). Briefly, 5 mg of digested protein per sample were solubilized in a 300 mg/ml lactic acid solution at pH 3 and passed over the TiO_2_ resin. Unbound peptides were washed from the resin with 80% acetonitrile and 0.1% TFA, and phosphopeptides were eluted with 1% ammonium hydroxide. Phosphopeptide eluate was dried under vacuum and then solubilized for analysis in 25 μl 0.1% formic acid in water. LCMS analysis was performed using the Exigent NanoLC Ultra 2D system with the cHiPLC that was coupled to the AB SCIEX 5500 QTRAP mass spectrometer. Sample analysis used a trap-elute system in which a 4 μl sample volume was injected onto a 200 μm × 6 mm ChromXP C18-CL, 3 μm, 120 Å trapping column (AB Sciex) running 0.1% formic acid at 0.5 μl/min. Analytical separation was performed on a 75μm × 15cm analytical chip column containing the same stationary phase as the trapping column and using a linear gradient of 0% to 30% acetonitrile with 0.1% formic acid for 70 min at a flow rate of 300 nl/min. The amount of phosphopeptide enriched from each sample was not quantified; however, the major advantage of using stable-isotope labeled standards added prior to digestion and subsequent sample handling steps is that small variations in phosphopeptide enrichment and instrument performance will affect both the labeled standard and the endogenous peptides equally, enabling comparisons across different samples by expressing peptide abundances as a ratio of endogenous peptide to labeled standard peptide. Each peptide was monitored with an eight-minute-wide chromatographic window that was centered at the elution time of the heavy-isotope standard. Three technical replicate injections were performed for each biological replicate sample. SRM transitions were optimized in Skyline version 2.6.06709 using synthetic heavy-isotope-labeled standards that were modified at the serine or threonine residue as determined in previous discovery studies on Orbitrap mass spectrometers. The most intense transitions consisting of a doubly or triply-charged precursor and singly- or doubly-charged fragments were selected from the b- or y-type ions. Occasionally, higher charge states were monitored for the longer peptides. These transitions were further optimized to empirically determine the best collision energy for detection. Ultimately, the most intense three to five transitions were used for subsequent analysis. The monitored masses appear in S1 Table. In general, y-ions were preferred for use both for quantitation and qualification (i.e. validation). However, in some cases the strongest signals were obtained from b-ions. This is possible because although the fragment ions from endogenous and labeled standards may have the same *m/z* value, the precursor masses still differ due to the presence of the heavy isotope label. This ensures that there is no crosstalk between heavy and light channels, even when the fragment ion mass is the same. The data were manually analyzed using at least 2 transitions per peptide for peak validation. One transition was chosen for quantitation while the other was used for qualification. Peak areas were integrated using Skyline. The mean peak area ratio, of multiple injection replicates (technical replicates) was calculated to arrive at a ratio value for each of the five biological replicates. These means were then used to calculate a mean for samples comprising each treatment, time point and genetic group. Finally, the experimentally-treated-to-control ratio and *p*-value were calculated (two tailed Student's *t*-test assuming equal variance) using Excel 2010.

The mass spectrometry proteomics data were deposited to the PASSEL [34] via the Peptide Atlas repository maintained by the Institute of Systems Biology (http://www.peptideatlas.org/passel/) with the dataset identifier PASS00816.

## Results

In our prior work [26], an isobaric tagging strategy was used to probe the effects on phosphorylation of Nod factor treatment in wild type and mutant plants grown by different conditions (hydroponic, aeroponic, and plate-based) and across several time points, This earlier work used a combination of isobaric tagging reagents and fragmentation mechanisms, including ETD, CID, and HCD. In order to streamline the quantitation of these phosphorylation events across multiple time points, mutant genotypes, and biological replicates, we transitioned to an SRM-based targeted strategy. Approximately 80 phosphopeptides were initially chosen from these results for inclusion. Many of these peptides were not observable due to changes in hydrophobicity or their performance on the triple-quadrupole system (as opposed to the previous Orbitrap system). Twenty phosphopeptides originating from this earlier study [26] were reliably detectable with good chromatographic performance. Additionally, twenty-five phosphorylation sites were chosen based on their established role in symbiotic signaling. Preliminary studies showed that phosphopeptide enrichment was required to observe signals for these targets from unfractionated digested protein extracts. From this group of forty-five, fifteen endogenous phosphopeptides were consistently detectable in all genetic backgrounds, treatments and time points at quantifiable levels (S2 Table). Within the first hour, five phosphopeptides, Medtr5g008900 (Zinc finger CCCH), Medtr7g068220 (Unknown function), Medtr8g104290 (Unknown function), Medtr6g012990, Sucrose non-fermenting1-related kinase (SNF1-related Kinase), and Medtr4g127710 (Proton ATPase MtAHA4), displayed an increase in phosphorylation after NF treatment in the wild-type, with the most significant changes occurring at approximately 15 min (S2 Table).

**Fig 1:**
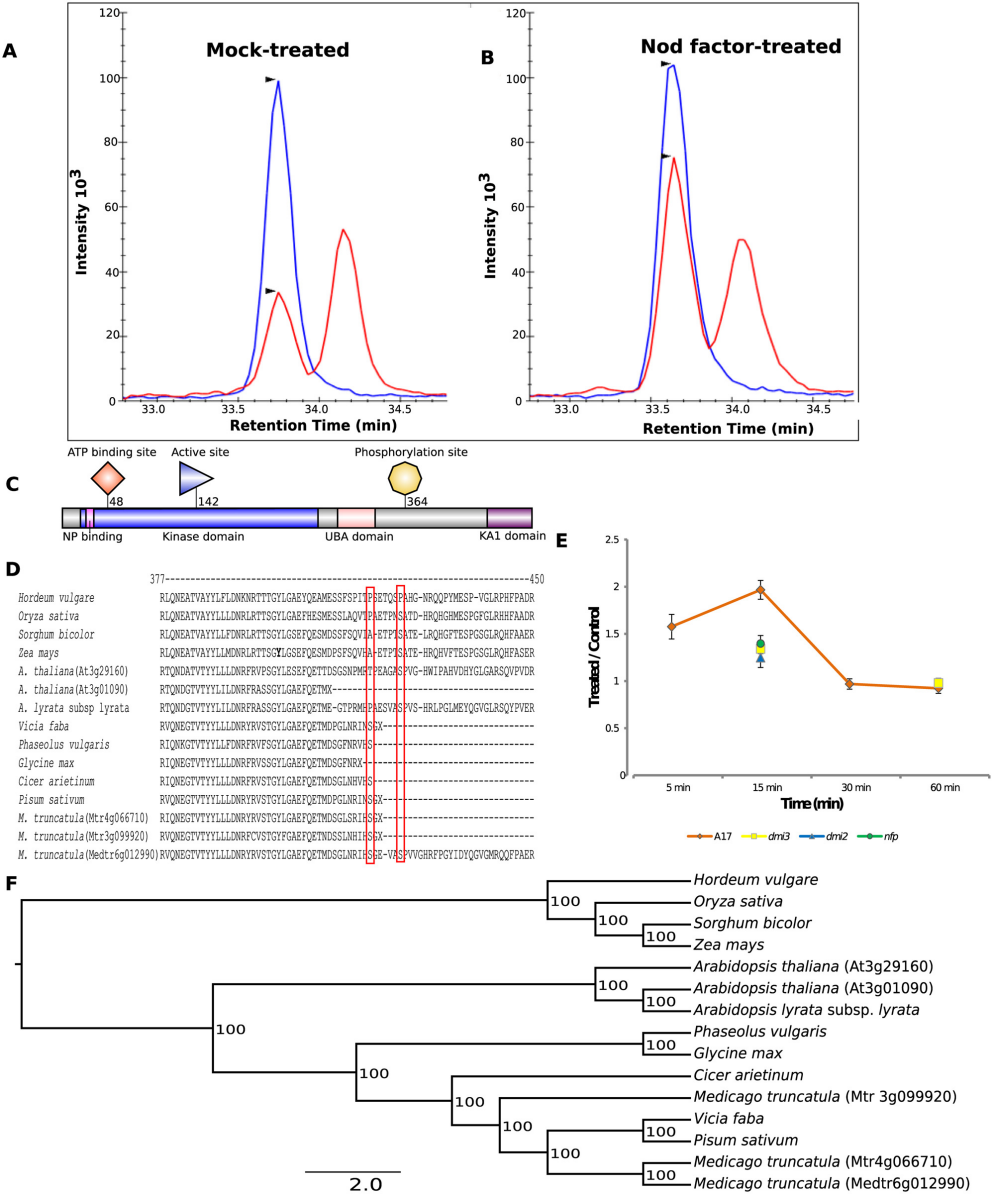
Phosphorylation of SNF1-related kinase (Medtr6g01290) upon treatment with NF. A) Representative chromatograms for SNF1-related kinase in the mock-treated samples. Blue trace corresponds to the heavy standard, and red corresponds to the light endogenous peptide. B) SNF1 showed changes in phosphorylation for site s364 after 15 min of treatment with NF in wild-type seedlings. Two closely related phosphopeptides (s359 and s364) were measured in all samples. C) Protein domain structure of the SNF1-related kinase. Only site s364, which showed significant phosphorylation in wild-type seedlings, is represented here. D) Amino acid sequence alignment of various plant SNF1-related kinases. Red box indicates the two phosphorylation sites s359 and s364. Site s359 is specific to legumes except for *Glycine max*, whereas site s364 is conserved in legumes and non-legumes, with the exception of *Hordeum vulgare*. E) One-hour time course to monitor SNF1-related kinase phosphorylation in wild-type seedlings, along with the 15-min time point for *nfp*, *dmi2* and *dmi3* mutants. In addition, for the *dmi3* mutants, the phosphorylation changes were also monitored at 60 min. A 2-fold increase in phosphorylation was observed in NF-treated wild-type (A17) seedlings within the first 15 min; by 30 min, there was no difference between the treated and control samples. Additionally, at 15 min, all three mutants clustered together near a 1.3-fold increase in phosphorylation in treated seedlings. Error bar represents standard error of mean. F) Phylogenetic analysis of the amino acid sequences of SNF1-related kinases using Mr. Bayes method. The analysis indicates that monocots clustered together, and within the dicots, the non-symbiotic Arabidopsis plant formed a separate clade. *M*. *truncatula* clustered together with the other legumes.

In our discovery study, SNF1-related kinase that was phosphorylated at s364 exhibited a nearly 2-fold increase in phosphorylation in the wild-type, while the three mutants varied between 1.25 to 1.4 fold at the 15-minute treatment time (S2 Table, Fig 1E). By 30 min, there was no measurable difference in phosphorylation between the treated and control wild-type plants (Fig 1E). SNF1-related proteins are evolutionarily conserved serine/threonine kinases that have primarily been implicated in starch and carbohydrate metabolism in plants [35]. In addition to their role in regulating energy metabolism, these proteins have recently been considered as the hub of the transcription network for stress and energy signaling [35]. Furthermore, SNF1-related kinases have been implicated in ABA signaling and in the direct phosphorylation and inactivation of various enzymes, such as HMG-CoA reductase, sucrose phosphate synthase, nitrate reductase, and trehalose-phosphate synthase. HMG-CoA reductase is a key enzyme in the mevalonate pathway of isoprenoid biosynthesis. In *M*. *truncatula*, a HMG-CoA reductase interacts with the receptor-like kinase DMI2 and is essential for early NF signaling and nodulation [36]. In addition, ABA signaling impacts NF signaling, nodule development and nitrogen fixation. Taken together, we hypothesize that SNF1-related proteins may impact NF signaling through the isoprenoid pathway or the ABA signaling pathway.

Protein sequences of SNF1-related kinase were aligned using MUSCLE (http://www.ebi.ac.uk/Tools/msa/muscle/), and the alignment was curated using BioEdit [37]. These sequences were subjected to phylogenetic analysis using Mr. Bayes [38]. Phylogenetic analysis revealed that SNF1-related kinases from all of the monocots, (*Oryza sativa*, *Hordeum vulgare*, *Sorghum bicolor* and *Zea mays*) clustered together whereas the dicots, including non-symbiotic Arabidopsis *thaliana* (Arabidopsis), formed a separate clade (Fig 1F). It is interesting to note that within the dicots, all of the Arabidopsis SNF1-related kinases constituted a clade different from the legume SNF1-related kinase clade suggesting that these legume SNF1-related kinases might have evolved to perform other legume specific functions, a situation analogous to the proposed cereal specific SnRK1b [39]. An analysis of the *M*. *truncatula* SNF1-related kinase (Medtr6g012990) protein domain structure revealed that this protein contains a kinase domain at the N-terminus and a regulatory domain at the C-terminus analogous to yeast, mammalian and other plant SNF1-related kinases [39, 40] (Fig 1C). Furthermore, Thr-172 of mammalian ortholog AMP-activated protein kinase (AMPK) and yeast ortholog SNF1 (represented by Thr-210), implicated in the activation of this protein by phosphorylation is conserved in Medtr6g012990 (Thr-175) along with its surrounding conserved region [39, 41]. This supported our hypothesis that *M*. *truncatula* SNF1-related kinase may also be regulated by phosphorylation. Because we identified that site s364 was phosphorylated in a NF dependent manner in the discovery study, we designed and synthesized heavy isotope labeled peptides to monitor this site by SRM. In close proximity to s364 another phosphorylated site, s359, is present. Sequence alignment revealed that this site (s359) is conserved across legume plants except for *Glycine max*, and we hypothesize that this site may play a role in legume specific function (Fig 1D). Furthermore, it is interesting to note that the site s364 is conserved across legumes and non-legumes (except for *Hordeum vulgare)* and that this site exhibited increased phosphorylation both in our previous discovery study and in our current targeted study. At this point, the role of this conserved site is not known However, since this site is conserved across plants (both symbiotic and non-symbiotic), it is reasonable to assume that it may play a role in the regulation of SNF1-related kinases.

**Fig 2:**
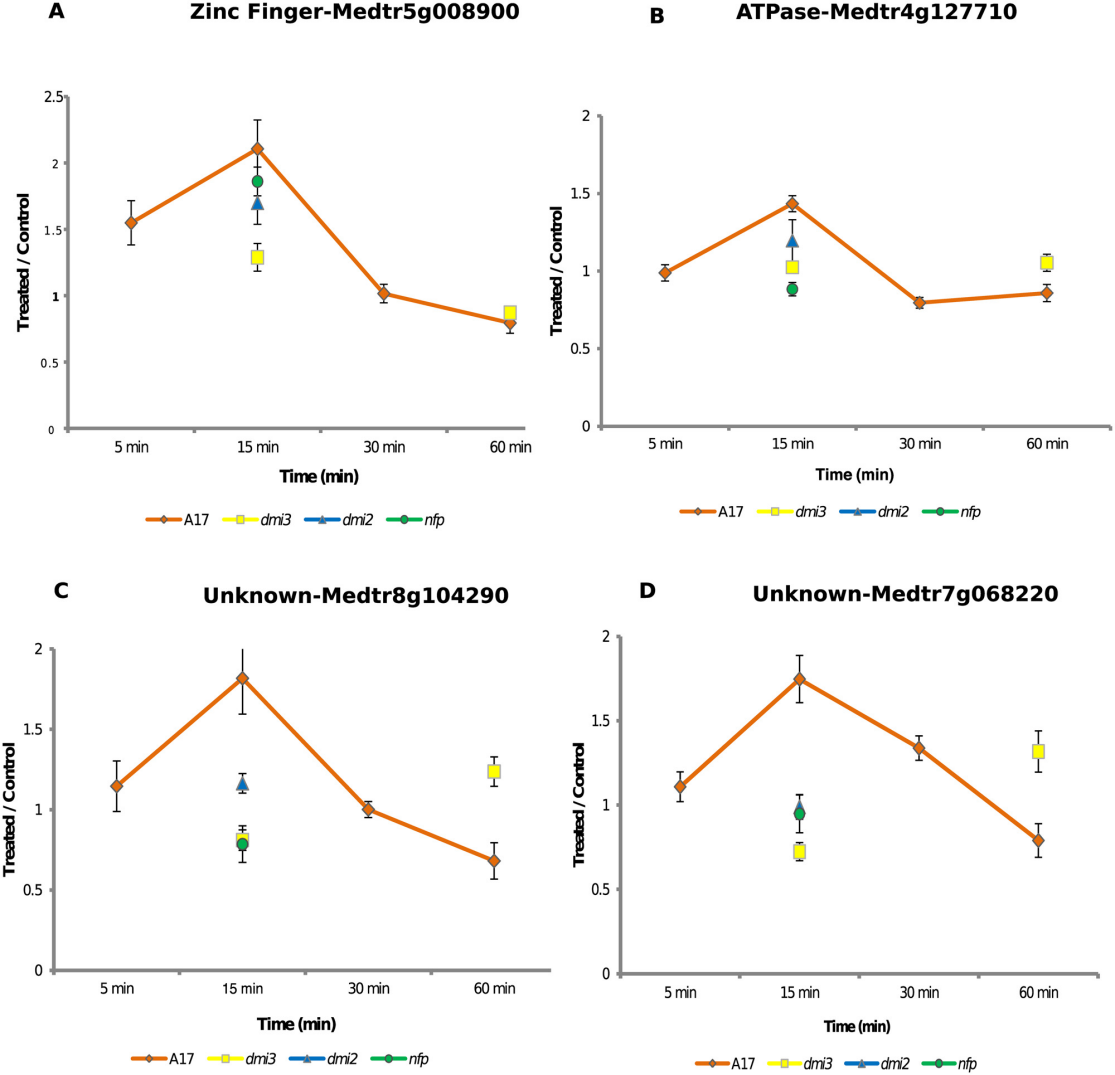
Phosphorylation of other peptides that exhibited significant differences upon treatment with NF. A) Zinc finger peptide displays a similar response profile as that of SNF1-related kinase to NF treatment in wild-type seedlings in the first 30 min; by 60 min, the treated seedlings begin to exhibit a decrease in phosphorylation. In the mutant seedlings at 15 min the peptide displays a differential phosphorylation decrease with NF treatment compared to the wild-type treated with Nod factors. B) Phosphorylation of the s8 residue of MtAHA4 increases significantly at the 15-minute sampling point and then decreases significantly at 30 min in wild-type seedlings. C) This peptide was derived from the hypothetical protein Medtr8g104290 of unknown function. NF treatment results in significant increase in phosphorylation at this site in wild-type seedlings at 15 min. D) Similar to Medtr8g104290, the peptide that derived from Medtr7g068220 (Unknown function) also exhibited a significant change in phosphorylation level at the 15-min time point for the wild-type in comparison to the mutants. Error bar represents standard error of mean for all these figures.

Zinc finger proteins belong to one of the largest family of transcription factors; in particular, zinc fingers with CCCH domains are a special class of transcription factors that directly bind to mRNA [42]. The CCCH family of zinc finger proteins has been implicated not only in plant responses to biotic and abiotic stresses, but also in plant development and adaptive processes [42]. In *M*. *truncatula*, at least 34 members of this family have been identified so far, although their role in legume symbioses has not been well-characterized. In our current work, the phosphorylation of the zinc finger peptide in wild-type seedlings increased more than 1.55 fold at 5 min and 2.1-fold at 15 min with NF treatment compared to the mock-treated control group (S2 Table). All of the treated mutant plants (except for *dmi3*) demonstrated a significant increase in phosphorylation for this peptide at 15 min, although not to the same level as the wildtype. The *dmi3* mutant exhibited the smallest change, with a 1.29-fold increase (Fig 2A).

Besides these two proteins, the plasma membrane proton pump MtAHA4 (Medtr4g127710) displayed increased phosphorylation to 1.43 fold at 15 min in wild-type seedlings (Fig 2B). In the *nfp*, *dmi2* and *dmi3* mutants at the same time point, the fold change was insignificant (S2 Table). By the 30-minute time point, the wild-type phosphorylation decreased to 0.80 compared to that of the mock-treated group (*p*-value 0.004). The characterization of MtAHA4 provided in this targeted analysis may provide new insights into the role of proton ATPases in the legume—rhizobium association. The second plasma membrane pump MtAHA5 (t957, Medtr2g036650) that was phosphorylated at the penultimate threonine residue showed no changes in the phosphorylation level in the wild-type between treated and control seedlings at any point in the time course in this targeted analysis. In the previous discovery experiments, MtAHA5 showed NF—dependent changes although there was considerable variability in the quantitation between biological replicates and the changes were not statistically significant. The role of other plasma membrane ATPases in symbiosis has been established mostly in the arbuscular mycorrhizal symbiosis. For instance, the plasma membrane proton ATPase MtHA1 is essential for the development of arbuscules in roots that are colonized with arbuscular mycorrhizal fungi [43].

Phosphorylation of peptides from two hypothetical proteins, s142 of Medtr8g104290 and s277 of Medtr7g068220 (Fig 2C and 2D), were identified in the discovery study as decreasing in phosphorylation at 60 min and showed a similar response in this study. The response pattern for both phosphopeptides is similar across the time course, with an increase in phosphorylation with treatment of approximately 1.8–fold at 15 min in wild-type (*p*-value 0.001 for s277 and 0.0039 for s142 at 15 min in wild-type), with mutants showing little change. At 60 min, in the *dmi3* mutant this peptide is phosphorylated at a much higher level than is the wild-type; however, the data fall below statistical significance (*p*-value 0.09). Other phosphopeptides with significant differences between the treated and control (t/c) were Medtr4g128650 (Pkinase-like, wild-type—15 min, 1.24 t/c, *p*-value 0.017; *dmi3*–15 min, 0.88 t/c, *p*-value 0.02; wild-type—60min, 0.87 t/c, *p*-value 0.023), Medtr7g085800 (Tubulin, *nfp*—15 min, 0.79 t/c, *p*-value 0.009), Medtr3g030040 (Cellulose synthase, *dmi3*–15 min, 0.83 t/c, *p*-value 0.010), and Medtr2g437530 (Uncharacterized, *dmi2*—15min, 1.17 t/c, *p*-value 0.014; *dmi3*—60min, 0.69 t/c, *p*-value 0.001) (S2 Table).

Most of the significant changes in the phosphorylation level occurred 15 min after treatment with Nod factors. This time point coincides with nuclear calcium spiking, a central event in symbiotic signaling that also begins approximately 15 min after the application of Nod factors. Taken together this result suggests that these proteins are prime candidates for transducing signals from the plasma membrane to the nucleus. After 15 min, there is generally a decrease in phosphorylation, which we speculate may be due to the rapid turnover of proteins or to physical interaction with phosphatases that degrade the kinase-mediated signal. For instance, SNF1-related protein kinase1 of Arabidopsis interacts with PP2CA phosphatases [44]. Similarly, members of another SnRK subfamily member in Arabidopsis (SnRK2) that have been implicated in ABA signaling interact with PP2CA phosphatases [31].

## Discussion

There are two paradigms for quantitative mass spectrometric measurements of phosphorylation currently in use: (1) untargeted, discovery measurements using high resolution instruments (e.g., Orbitrap) and (2) targeted analyses by SRM on triple quadrupole instruments or PRM (parallel reaction monitoring) on high-resolution, MS/MS-capable instruments (e.g. Q-Exactive or Q-TOF). While the discovery-based system allows measurements of tens of thousands of proteins and the targeted method at most, only hundreds, the increased number of proteins analyzed comes at the expense of sensitivity. Thus, for maximum sensitivity and dynamic range, quadrupole- based targeted analyses using SRM is the method of choice.

SRM relies on the ability to routinely and reproducibly detect a desired peptide with limited pre-fractionation aided by the presence of a synthetic heavy-isotope-labeled 'spiked' internal standard. The principles of SRM and targeted quantitative proteomics have been reviewed extensively (e.g. [45] and [46]) and so will not be covered here. Although SRM-based quantitation of proteins in plants is common [47–55], there are fewer examples of its use in quantitative plant phosphoproteomics [31, 32, 56–58]. Fewer still are the instances where discovery-based phosphoproteomic approaches have been validated by follow-up targeted work [31, 32]. Targeted measurement of individual sites of protein phosphorylation by SRM (i.e. specific phosphopeptides) brings additional challenges compared to quantitation at the protein level for several reasons. Among these are: the choice of peptides to monitor as proxies for protein abundance are not optimizable; the stoichiometry of phosphorylation tends to be low; and the post-translational modification is both chemically and enzymatically labile. Furthermore, the location of the phosphorylated residue can be difficult to verify from spectral data in cases where multiple modifiable residues are present in a single tryptic peptide, and each of these phosphosites in a peptide produces slightly different retention characteristics via HPLC.

Here, we used targeted SRM to measure changes in the phosphorylation of *M*. *truncatula* seedlings at four time points during the first hour of the NF response using wild-type seedlings and mutants that are critical to the NF signaling pathway. Treated and control samples at each time point were processed in parallel from start to finish. Five biological replicates for each condition were included for statistical analyses. Generally, the coefficients of variation between biological replicates were less than 20%. The signal intensity of fragment ions depends on the amino acids constituting that fragment. The charged amino acids histidine, lysine, arginine and proline result in the best (highest intensity) fragment ions for detection, but these ions are not always the most informative. Often, there are several possible sites of phosphorylation within a tryptic fragment. Precursor mass and highest intensity fragment masses may be the same regardless of the location of the phosphorylation. Only fragmentation between two possible sites will enable the localization of the phosphorylated residue, and the closer the two sites, the more challenging it is to make a confident determination. The different sites of phosphorylation usually result in a chromatographic retention time shift, but the precursor and monitored product ion masses can be identical. For several peptides in this study, multiple endogenous phosphorylation sites were measured simultaneously, for the reasons described above- precursor and product ion masses are constant, but the different phosphopeptides are chromatographically resolved. In these cases, one endogenous peak co-eluted with the isotopic standard while the other shifted within the chromatographic window. One such case, MtAHA4, was detected as two chromatographically resolved endogenous phosphopeptides. The width of the scheduling window was fortuitous, because a smaller window for this peptide would have prevented detection of the alternately phosphorylated form. Similarly, two distinct phosphorylation sites were observed on a single tryptic peptide for SNF1-related kinase (Fig 1A and 1B) with narrow but clear chromatographic resolution. Fortunately, in both of these cases the peptide sequences included only two possible sites of phosphorylation. One of these sites co-eluted with the synthetic isotopic standard, confirming the assignment of the phosphorylation site for that peptide, and thus determining the phosphorylation site on the alternate peptide as well. In an attempt to confirm the phosphate group localization, an SRM-triggered enhanced product ion (EPI) scan, using the linear ion trap functionality of the mass spectrometer, was performed on a treated 15-minute sample. This type of acquisition continuously scans for specific pairs of precursor and fragment ions. Whenever the pair is detected, the mass spectrometer will switch modes from selected-ion to full-spectrum acquisition. The spectra that were produced from this scan confirmed the phosphorylation site for both SNF1-related kinase and the ATPase MtAHA4. Both sites were detected in the discovery study, but only one of the sites displayed a significant change in occupancy. Three phosphopeptides targets (Zinc finger, Medtr2g437530, and lipoxygenase) had no measurable levels of endogenous phosphopeptide co-eluting with the isotopic standard, although other endogenous peaks were present with a slightly shifted retention time (~0.3 min). The Zinc finger peptide (NPSSPsGNVWSQPSFPK) has five possible phosphorylation sites, two of which (s106 and s108) were detected in the discovery study, with s108 showing a change with NF treatment. The retention time of the endogenous phosphopeptide observed in this study was shifted relative to that of s108, for which the phosphorylated heavy-isotope standard was included in all samples. The relative intensity and transition order between the standard and endogenous peaks were identical. In this case, the specific phosphorylated residue could not be verified using the QTRAP EPI scan method, so differentially phosphorylated standards were synthesized to verify the location of the endogenous phosphorylation, confirming the correct location at s106. In addition, phosphorylated standards were also synthesized for target Medtr2g437530 (VEEAPATTETKTEDEtSGVK), showing that the change in phosphorylation upon NF stimulation occurred at t 147 rather than t 151, as previously concluded based on discovery data.

## Supporting Information

S1 Table List of phosphopeptides used for targeted SRM analysis and their transitions used for quantification. (DOCX)

S2 Table Experimental results for the phosphopeptides analyzed using SRM at different time points after NF treatment in wild-type and mutant seedlings. (DOCX)
