# Supplementary material for: Mass Spectrometric-Based Selected Reaction Monitoring of Protein Phosphorylation during Symbiotic Signaling in the Model Legume, Medicago truncatula
Source: PLoS One. 2016 May 20;11(5):e0155460. doi: 10.1371/journal.pone.0155460 (PMC4874550; doi:10.1371/journal.pone.0155460)
Supplement: S1 Table — (DOCX) [file pone.0155460.s001.docx]

**S1Table: List of phosphopeptides used for targeted SRM analysis and their transitions used for quantification**

| **Gene ID** | **Peptide Sequence** | **Phospho Site** | **Name** | **Precursor Charge** | **Light Precursor *m/z*** | **Light Fragment** | **Heavy Precursor *m*/*z*** | **Heavy Fragment** | **CE** | **DP** |
| --- | --- | --- | --- | --- | --- | --- | --- | --- | --- | --- |
| Medtr3g030040 | MSMAsPVNAR | s165 | CELL SYTHASE  Like | 2 | **572.238** | **556.32/y5+** | **574.242** | **560.327/y5+** | 72.8 | 29.5 |
|  |  |  |  |  |  | 794.356/y7+ |  | 798.363/y7+ |  |  |
|  |  |  |  |  |  | 463.202/y8++ |  | 465.205/y8++ |  |  |
| Medtr8g056900 | VNSQLSsPSQPYSLR | s1306 | Guanidine  Nucleotide  Exchange  factor | 2 | **871.909** | **635.351/y5+** | **875.418** | **642.368/y5+** | 94.7 | 40.2 |
|  |  |  |  |  |  | 947.495/y8+ |  | 954.512/y8+ |  |  |
|  |  |  |  |  |  | 850.442/y7+ |  | 857.459/y7+ |  |  |
|  |  |  |  |  |  | 375.235/y3+ |  | 382.252/y3+ |  |  |
| Medtr5g027010 | TDsEELFK | s50 | Trans  membrane  protein,  putative | 2 | **524.715** | **849.399/y7-98+** | **528.722** | **857.413/y7-98+** | 69.4 | 27.7 |
|  |  |  |  |  |  | 734.372/y6-98+ |  | 742.386/y6-98+ |  |  |
|  |  |  |  |  |  | 407.265/y3+ |  | 415.279/y3+ |  |  |
|  |  |  |  |  |  | 294.181/y2+ |  | 302.195/y2+ |  |  |
| Medtr8g018520 | SEsRSDFVYIPR | s211 | Lipoxy | 3 | **512.568** | **548.319/y4+** | **514.572** | **554.333/y4+** | 68.5 | 25.5 |
|  |  |  |  |  |  | 385.256/y3+ |  | 391.27/y3+ |  |  |
|  |  |  |  |  |  | 272.172/y2+ |  | 278.186/y2+ |  |  |
|  |  |  |  |  |  | 889.309/b7+ |  | 889.309/b7+ |  |  |
|  |  |  |  |  |  | 791.332/b7-98+ |  | 791.332/b7-98+ |  |  |
| Medtr2g437530.1 | VEEAPATTETKTEDEtSGVK | t151 | m put t151 | 3 | **734.661** | **358.161/b3+** | **736.666** | **358.161/b3+** | 84.7 | 37.6 |
|  |  |  |  |  |  | 987.433/y18++ |  | 990.44/y18++ |  |  |
|  |  |  |  |  |  | 887.393/y16++ |  | 890.4/y16++ |  |  |
|  |  |  |  |  |  | 838.867/y15++ |  | 841.874/y15++ |  |  |
| Medtr4g127710 | sISLEQIK | s6 | atpase  (MtHA4) | 2 | **499.252** | **517.298/y4+** | **502.76** | **524.315/y4+** | 67.5 | 26.8 |
|  |  |  |  |  |  | 630.382/y5+ |  | 637.399/y5+ |  |  |
|  |  |  |  |  |  | 388.255/y3+ |  | 395.273/y3+ |  |  |
|  |  |  |  |  |  | 260.197/y2+ |  | 267.214/y2+ |  |  |
| Medtr2g036650 | GLDIETMQQHYtV | t957 | atpase  (MtHA5) | 2 | **807.847** | **286.14/b3+** | **810.854** | **286.14/b3+** | 90.0 | 37.9 |
|  |  |  |  |  |  | 989.451/y8-98+ |  | 995.465/y8-98+ |  |  |
|  |  |  |  |  |  | 399.224/b4+ |  | 399.224/b4+ |  |  |
| Medtr4g128650 | TSLVEYPSsPSGPR | s282 | Kinase  Super  family | 2 | **778.853** | **864.361/y8+** | **781.86** | **870.375/y8+** | 87.9 | 36.9 |
|  |  |  |  |  |  | 929.448/y9-98+ |  | 935.461/y9-98+ |  |  |
|  |  |  |  |  |  | 766.384/y8-98+ |  | 772.398/y8-98+ |  |  |
|  |  |  |  |  |  | 513.278/y5+ |  | 519.292/y5+ |  |  |
| Medtr2g087820 | sPNQSSPSSQDR | s246 | ring43 s246 | 2 | **685.273** | **281.124/b3-98+** | **688.28** | **281.124/b3-98+** | 81.1 | 33.5 |
|  |  |  |  |  |  | 991.444/y9+ |  | 997.458/y9+ |  |  |
|  |  |  |  |  |  | 863.385/y8+ |  | 869.399/y8+ |  |  |
|  |  |  |  |  |  | 689.321/y6+ |  | 695.335/y6+ |  |  |
| Medtr8g086300 | SVsPPK | s295 | Scar 3- like s295 | 2 | **347.662** | **410.24/y4-98+** | **350.669** | **416.254/y4-98+** | 56.5 | 21.4 |
|  |  |  |  |  |  |  |  |  |  |  |
|  |  |  |  |  |  | 298.674/P-98+ |  | 301.681/P-98+ |  |  |
|  |  |  |  |  |  | 509.308/y5-98+ |  | 515.322/y5-98+ |  |  |
|  |  |  |  |  |  | 508.217/y4+ |  | 514.231/y4+ |  |  |
|  |  |  |  |  |  | 341.218/y3+ |  | 347.232/y3 |  |  |
| Medtr6g012990 | IHSGEVAsPVVGHR | s364 | snf1 s364 | 3 | **508.915** | **251.15/b2+** | **510.92** | **251.15/b2+** | 68.2 | 25.3 |
|  |  |  |  |  |  | 468.268/y4+ |  | 474.282/y4+ |  |  |
|  |  |  |  |  |  | 588.81/y12-98++ | | 591.817/y12-98++ |  |  |
| Medtr8g024050 | ILsSLSR | s49 | sol inorganiv  pyr s49 | 2 | **428.22** | **531.289/y5-98+** | **431.729** | **538.306/y5-98+** | 62.3 | 24.3 |
|  |  |  |  |  |  | 644.373/y6-98+ |  | 651.39/y6-98+ |  |  |
|  |  |  |  |  |  | 629.265/y5+ |  | 636.283/y5+ |  |  |
|  |  |  |  |  |  | 462.267/y4+ |  | 469.284/y4+ |  |  |
| Medtr7g085800 | TVQFVDWCPtGFK | t49 | tua3 | 3 | **555.577** | **629.269/y5+** | **558.92** | **639.297/y5+** | 70.2 | 26.8 |
|  |  |  |  |  |  | 789.3/y6+ |  | 799.327/y6+ |  |  |
|  |  |  |  |  |  | 532.217/y4+ |  | 542.244/y4+ |  |  |
|  |  |  |  |  |  | 575.319/b5+ |  | 575.319/b5+ |  |  |
| Medtr8g104290 | SNsTGSAPNLK | s142 | unk mt8 s142 | 2 | **578.256** | **686.383/y7+** | **581.764** | **693.4/y7+** | 73.3 | 29.7 |
|  |  |  |  |  |  | 787.431/y8+ |  | 794.448/y8+ |  |  |
|  |  |  |  |  |  | 629.362/y6+ |  | 636.379/y6+ |  |  |
| Medtr7g068220 | GIsPSVK | s277 | unk mt7 s277 | 2 | **384.189** | **597.264/y5+** | **387.195** | **603.278/y5+** | 59.1 | 22.7 |
|  |  |  |  |  |  | 430.266/y4+ |  | 436.28/y4+ |  |  |
|  |  |  |  |  |  | 333.213/y3+ |  | 339.227/y3+ |  |  |
| Medtr5g008900 | NPSSPsGNVWSQPSFPK | s108 | zinc finger s108 | 3 | **632.616** | **575.319/y5+** | **634.62** | **581.333/y5+** | 77.2 | 32.0 |
|  |  |  |  |  |  | 976.489/y8+ |  | 982.503/y8+ |  |  |
|  |  |  |  |  |  | 790.409/y7+ |  | 796.423/y7+ |  |  |
|  |  |  |  |  |  | 703.377/y6+ |  | 709.391/y6+ |  |  |
|  |  |  |  |  |  | 920.351/b9+ |  | 920.351/b9+ |  |  |
