# Supplementary material for: Mass Spectrometric-Based Selected Reaction Monitoring of Protein Phosphorylation during Symbiotic Signaling in the Model Legume, Medicago truncatula
Source: PLoS One. 2016 May 20;11(5):e0155460. doi: 10.1371/journal.pone.0155460 (PMC4874550; doi:10.1371/journal.pone.0155460)
Supplement: S2 Table — (DOCX) [file pone.0155460.s002.docx]

**S2 Table: Experimental results for the phosphopeptides analyzed using SRM at different time points after NF treatment in wild-type and mutant seedlings.**

| **Gene ID** | **5 min** | | | **15 min** | | | | | | | | | | | | **30 min** | | | **60 min** | | | | | |
| --- | --- | --- | --- | --- | --- | --- | --- | --- | --- | --- | --- | --- | --- | --- | --- | --- | --- | --- | --- | --- | --- | --- | --- | --- |
|  | Wild-type (A17) | | | Wild-type (A17) | | | *nfp* | | | *dmi2* | | | *dmi3* | | | Wild-type (A17) | | | Wild-type (A17) | | | *dmi3* | | |
|  | **T/C ratio** | **SE** | ***p-*value** | **T/C ratio** | **SE** | ***p-*value** | **T/C ratio** | **SE** | ***p-*value** | **T/C ratio** | **SE** | ***p-*value** | **T/C ratio** | **SE** | ***p-*value** | **T/C ratio** | **SE** | ***p-*value** | **T/C ratio** | **SE** | ***p-*value** | **T/C ratio** | **SE** | ***p-*value** |
| **Medtr3g030040**  **(Cellulose Synthase like protein)** | 0.89 | 0.12 | 0.53643 | 0.89 | 0.04 | 0.12287 | 1.18 | 0.17 | 0.45659 | 0.96 | 0.09 | 0.79667 | 0.83 | 0.03 | 0.01063 | 0.99 | 0.05 | 0.88072 | 0.93 | 0.06 | 0.48055 | 1.14 | 0.06 | 0.13684 |
| **Medtr8g056900**  **(Guanaine nucleotide exchange factor, putative)** | 1.03 | 0.04 | 0.65455 | 1.05 | 0.06 | 0.55540 | 0.92 | 0.03 | 0.11612 | 1.03 | 0.06 | 0.69849 | 1.02 | 0.05 | 0.80119 | 0.92 | 0.04 | 0.21597 | 0.95 | 0.03 | 0.37538 | 1.06 | 0.03 | 0.19232 |
| **Medtr5g027010**  **(transmembrane protein, putative)** | 0.87 | 0.09 | 0.39693 | 0.84 | 0.09 | 0.31643 | 1.18 | 0.07 | 0.09138 | 1.20 | 0.09 | 0.14352 | 0.90 | 0.11 | 0.55085 | 0.91 | 0.08 | 0.42686 | 1.02 | 0.10 | 0.86714 | 0.72 | 0.14 | 0.24291 |
| **Medtr8g018520**  **(9S-lipoxygenase)** | 1.01 | 0.04 | 0.81190 | 1.05 | 0.03 | 0.25532 | 0.96 | 0.04 | 0.49129 | 0.95 | 0.06 | 0.56994 | 0.97 | 0.02 | 0.47614 | 0.88 | 0.04 | 0.08877 | 0.90 | 0.05 | 0.19977 | 0.95 | 0.07 | 0.60808 |
| **Medtr2g437530.1**  **(Putative Unchar)** | 0.96 | 0.05 | 0.58395 | 1.06 | 0.06 | 0.47981 | 1.04 | 0.06 | 0.67029 | 1.17 | 0.04 | 0.01461 | 0.99 | 0.04 | 0.90317 | 1.00 | 0.06 | 0.96387 | 1.21 | 0.16 | 0.33051 | 0.69 | 0.04 | 0.00105 |
| **Medtr4g127710**  **(Plasma membrane proton ATPase, MtHA4)** | 0.99 | 0.05 | 0.88013 | 1.43 | 0.05 | 0.00015 | 0.88 | 0.04 | 0.09467 | 1.20 | 0.13 | 0.32861 | 1.02 | 0.03 | 0.57283 | 0.80 | 0.03 | 0.00413 | 0.86 | 0.06 | 0.12491 | 1.05 | 0.06 | 0.50710 |
| **Medtr2g036650**  **(Plasma membrane proton ATPase, MtHA5)** | 1.03 | 0.08 | 0.79486 | 0.98 | 0.06 | 0.82301 | 0.95 | 0.06 | 0.59091 | 1.01 | 0.03 | 0.79162 | 0.94 | 0.06 | 0.49255 | 0.92 | 0.04 | 0.21370 | 0.97 | 0.04 | 0.61243 | 1.16 | 0.06 | 0.07780 |
| **Medtr4g128650**  **(Kinase super-family protein)** | 1.07 | 0.06 | 0.39399 | 1.24 | 0.06 | 0.01791 | 1.08 | 0.06 | 0.40078 | 1.09 | 0.07 | 0.36738 | 0.88 | 0.03 | 0.02126 | 1.10 | 0.06 | 0.26098 | 0.87 | 0.03 | 0.02328 | 1.04 | 0.03 | 0.39406 |
| **Medtr8g086300**  **(SCAR3- like)** | 0.99 | 0.05 | 0.88302 | 1.07 | 0.08 | 0.50706 | 1.30 | 0.26 | 0.40807 | 0.98 | 0.03 | 0.64033 | 1.18 | 0.10 | 0.20683 | 0.94 | 0.06 | 0.52766 | 1.03 | 0.05 | 0.68329 | 0.80 | 0.07 | 0.15967 |
| **Medtr6g012990**  **(SNF-1 related)** | 1.58 | 0.13 | 0.00711 | 1.97 | 0.10 | 0.00001 | 1.40 | 0.08 | 0.00673 | 1.25 | 0.10 | 0.10904 | 1.34 | 0.05 | 0.00045 | 0.97 | 0.05 | 0.69882 | 0.92 | 0.05 | 0.36297 | 0.98 | 0.05 | 0.77638 |
| **Medtr8g024050**  **(Sol Inorg Pyrophos)** | 1.15 | 0.08 | 0.21469 | 1.12 | 0.06 | 0.13493 | 0.90 | 0.07 | 0.38329 | 1.07 | 0.07 | 0.48587 | 0.96 | 0.04 | 0.53128 | 1.05 | 0.04 | 0.34836 | 0.95 | 0.04 | 0.32703 | 1.17 | 0.10 | 0.18197 |
| **Medtr7g085800**  **(Tubulin)** | 0.87 | 0.07 | 0.24409 | 0.88 | 0.06 | 0.20057 | 0.79 | 0.04 | 0.00951 | 0.89 | 0.10 | 0.50192 | 0.98 | 0.07 | 0.82948 | 0.93 | 0.05 | 0.35138 | 0.88 | 0.04 | 0.07380 | 1.08 | 0.10 | 0.55400 |
| **Medtr8g104290**  **(UNK chr8)** | 1.15 | 0.15 | 0.51293 | 1.82 | 0.22 | 0.00392 | 0.79 | 0.11 | 0.30101 | 1.16 | 0.06 | 0.08285 | 0.81 | 0.06 | 0.10531 | 1.00 | 0.05 | 0.98679 | 0.68 | 0.11 | 0.21134 | 1.24 | 0.09 | 0.09393 |
| **Medtr7g068220**  **(UNK chr7)** | 1.11 | 0.09 | 0.40121 | 1.75 | 0.14 | 0.00149 | 0.95 | 0.11 | 0.75173 | 0.99 | 0.07 | 0.91867 | 0.72 | 0.05 | 0.02482 | 1.34 | 0.07 | 0.00370 | 0.79 | 0.10 | 0.26017 | 1.32 | 0.12 | 0.09624 |
| **Medtr5g008900**  **(Zinc Finger)** | 1.55 | 0.17 | 0.02158 | 2.11 | 0.22 | 0.00035 | 1.86 | 0.11 | 0.00005 | 1.70 | 0.16 | 0.00597 | 1.29 | 0.10 | 0.06446 | 1.02 | 0.07 | 0.87533 | 0.79 | 0.08 | 0.12359 | 0.87 | 0.04 | 0.05199 |
